# Speech in action: degree of hand preference for grasping predicts speech articulation competence in children

**DOI:** 10.3389/fpsyg.2014.01267

**Published:** 2014-11-06

**Authors:** Claudia L. R. Gonzalez, Fangfang Li, Kelly J. Mills, Nicole Rosen, Robbin L. Gibb

**Affiliations:** ^1^The Brain in Action Laboratory, Department of Kinesiology, University of LethbridgeLethbridge, AB, Canada; ^2^Psychology, University of LethbridgeLethbridge, AB, Canada; ^3^Linguistics, University of ManitobaWinnipeg, MB, Canada; ^4^Neuroscience, University of LethbridgeLethbridge, AB, Canada

**Keywords:** grasp-to-eat, development, language, lateralization, hand-to-mouth

## Abstract

**Highlights**: Degree of lateralization for grasping predicts the maturity of the language production system in young, typically-developing children.

In this report we provide compelling evidence for the relationship between right hand grasp-to-mouth (i.e., feeding) movements and language development. Specifically, we show that children (4–5 years old) who are more right-hand lateralized in picking up small food items for consumption show enhanced differentiation of the “s” and “sh” sounds. This result suggests that left hemisphere control of hand-to-mouth gestures may have provided an evolutionary platform for the development of language. The current investigation presents the exciting possibility that early right hand-to-mouth training could accelerate the development of articulation skills.

## Introduction

Arguably, speech is the most complex human motor action, involving manipulations of about 100 vocal and respiratory muscles (Levelt, 1989). Numerous studies have shown the connection between fine motor skill and speech. For example, various speech disorders correlate with poor fine motor skills (Hill, 2001; Zelaznik and Goffman, 2010; Highman et al., 2013). Children with speech articulation deficits such as phonological disorder or developmental dyspraxia often present with deficits in manual dexterity (Viholainen et al., 2002; Visscher et al., 2007; Preston et al., 2010). This evidence suggests a common mechanism underlying both fine motor function and speech production. In the normally-developing population, however, evidence for this relationship is scarce. This is surprising given that the neural substrates that regulate motor control (in the frontal lobe) have been speculated to facilitate language acquisition in children (Iverson, 2010), as well as underlie the evolution of human language (Lieberman, 2002). In addition, neuroimaging studies have shown that Broca's area (in the frontal lobe) is involved in both speech production and non-linguistic motor tasks (for a review see Pulvermuller et al., 2005, 2006; Olivier et al., 2007). Despite the overwhelming evidence drawn from special populations and neuroimaging literature, motor development has been largely overlooked as a playmate of normal language development (Adolph et al., 2010).

The connection between fine motor control and speech functions is likely mediated by brain lateralization that biases specialization of these functions. For example, in patients with unilateral brain injury, high-level cognitive performance (including speech production and motor processing) is compromised in patients with left- but not right hemisphere damage (Barbey et al., 2012). More specifically, measures of general intelligence (some of which overlap with language) have been correlated with a left lateralized fronto-parietal network (Gläscher et al., 2010). Interestingly, the studies mentioned thus far all employed manual tasks as a measure of fine motor skill. This raises the possibility that the language and motor relationship is in fact a *language and hand relationship*. In neurologically-intact adult participants, it has been shown that the lateralization of hand use for grasping predicts the lateralization of language (Gonzalez and Goodale, 2009). In 13-month old babies right hand use correlated with analytic/receptive aspects of language development (Bates et al., 1986) and consistency of right hand use in babies was associated with advance language skills in toddlers (Nelson et al., 2014). Specific to speech, studies have shown that speech production is strongly lateralized to the left primary motor cortex (Wildgruber et al., 1996; Terumitsu et al., 2006; Brown et al., 2009), which controls the right hand. Using transcranial magnetic stimulation, it has shown that “motor structures [motor cortex] provide a specific functional contribution to the perception of speech sounds” (D'Ausilio et al., 2009). Regarding the hand, a recent study demonstrated that language comprehension activates hand specific regions in motor related brain structures (pre-motor cortex; Moody-Triantis et al., 2014). This evidence suggests that the relationship between cortical motor areas and speech goes beyond speech production to encompass speech comprehension. Shebani and Pulvermuller (2013) go even further to state “language and action systems of the human brain are functionally interwoven.” Taken together, these studies support the idea of an intimate relationship between lateralized hand function and speech articulation.

We are poised to directly test such a possibility. We hypothesize a relationship between right hand preference for grasping and proficiency of speech sound articulation in right-handed children. Specifically, articulation skills were assessed through the acoustic analysis of “s”-“sh” distinction (such as producing “sea” distinctively from “she”) in children's speech. The two sounds are (relatively) late-acquired sounds due to the motor demand of positioning the tongue tip with precision (Kent, 1992). Grasping performance was gauged through two tasks: picking up Lego blocks (grasp-to-construct) and food items (grasp-to-eat). Both tasks have been broadly used to determine lateralization for grasping in children (Sacrey et al., 2013; Gonzalez et al., 2014) and adults (Gonzalez and Goodale, 2009; Stone et al., 2013; Stone and Gonzalez, 2014). The grasp-to-eat task was of special interest as its use demonstrated that children develop a right-hand preference several years earlier than they do for grasp-to-construct task.

## Materials and methods

Thirty-five children were recruited from the Southern Alberta region through flyers placed at local elementary schools, libraries, recreational facilities, supermarkets, and toy stores. All children were identified as right-handed according to a modified version of the Edinburgh Handedness questionnaire (completed by each parent; see Stone et al., 2013) for full version of the questionnaire). Seventeen (10 females) were between 4 and 5 years of age and the remaining 18 (8 females) were between 8 and 9 years of age. The two age groups were selected to represent two developmental stages where 4–5 year olds are still in the process of learning to articulate “s” and “sh” while 8–9 year olds have already mastered the two sounds (Sander, 1972; Smit et al., 1990). Participants with disclosed neurological impairment or speech disorder were excluded from the study. The study was approved by the local ethics committee, and all caregivers gave written informed consent before their child participated in the study. Participants were naïve to the purposes of the study.

### Procedure

#### Speech

Children were recorded individually in a quiet room while seated in front of a computer monitor displaying a series of objects with names beginning with the “s” and “sh” sounds (/s/ and /ʃ/ in International Phonetic Alphabet respectively) while audio prompts were played simultaneously. These words were: *salad, salmon, sandwich, seahorse, seal, seat, soup, suit, suitcase, sheep, shadow, shoelace, shield, shallow, shoe, sheet, shack, and shoot*. Children played a game (Edwards and Beckman, 2008; Show and Play) in which a duck on the left margin of the screen climbs one step every time they speak a word into the microphone. The child's job was to help the duck climb to the very top of the ladder. The program has been used successfully in similar previous research on young children (Li et al., 2009; Li, 2012). Children's speech was recorded using a digital recorder (Marantz PMD 661) connected to a Shure SM87A condenser microphone placed directly in front of them and approximately 20 cm from their mouths. Recordings were made using a 44.1 kHz sampling rate and 16-bit quantization.

#### Acoustic analysis

**Figure 1:**
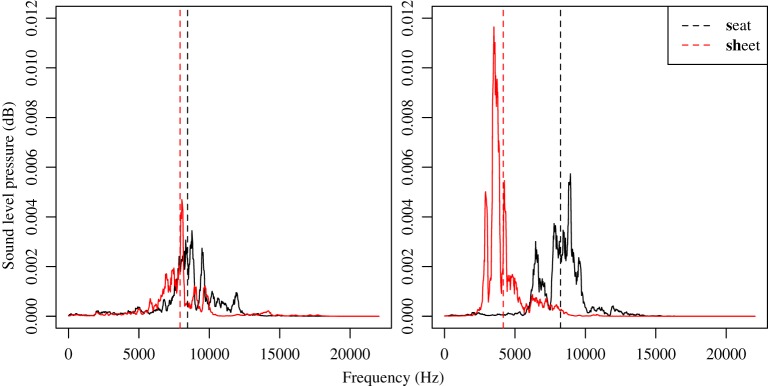
**Acoustic distance between “s” (in word *seat*) and “sh” (in word *sheet*) for a child who did not differentiate the two sounds (left) and one who clearly separated the two (right)**. Dotted lines indicate calculated spectral mean frequencies.

Digital spectrographic techniques were used to quantify children's speech production as they capture the gradience of articulatory gestures in speech that are otherwise elusive through auditory perception. In articulation, the main difference between “s” and “sh” lies in the relative tongue tip position in the oral cavity as well as the presence/absence of lip protrusion. The “sh” sound is produced with rounded lips and with a more posterior tongue position than the “s” sound (Ladefoged and Maddieson, 1996). Such articulatory difference can be characterized acoustically using the spectral mean frequency, which calculates the weighted mean frequency of the sound noise spectrum (Forrest et al., 1988; Jongman et al., 2000). The “sh” displays a lower overall energy distribution frequency in the noise spectrum, resulting in lower value of spectral mean. The degree of articulatory distinction between “s” and “sh” was assessed through the acoustic distance by taking the difference of spectral mean frequency between the two sounds. The greater the distance, the more distinctly their “s” and “sh” are articulated, and the more robustly the two sounds are contrasted (Figure 1).

The speech analysis software Praat Version 5.3.3.9 (Boersma and Weenink, 2013) was used for sound processing. Segments of “s” and “sh” were extracted and further processed through the Multitaper package (Rahim, 2010) in R (R Development Core Team, 2011). A spectrum based on a 40-ms slice surrounding the middle of each “s” and “sh” sound segment was made, from which spectral frequency value was calculated (Figure 1).

#### Grasping

**Figure 2:**
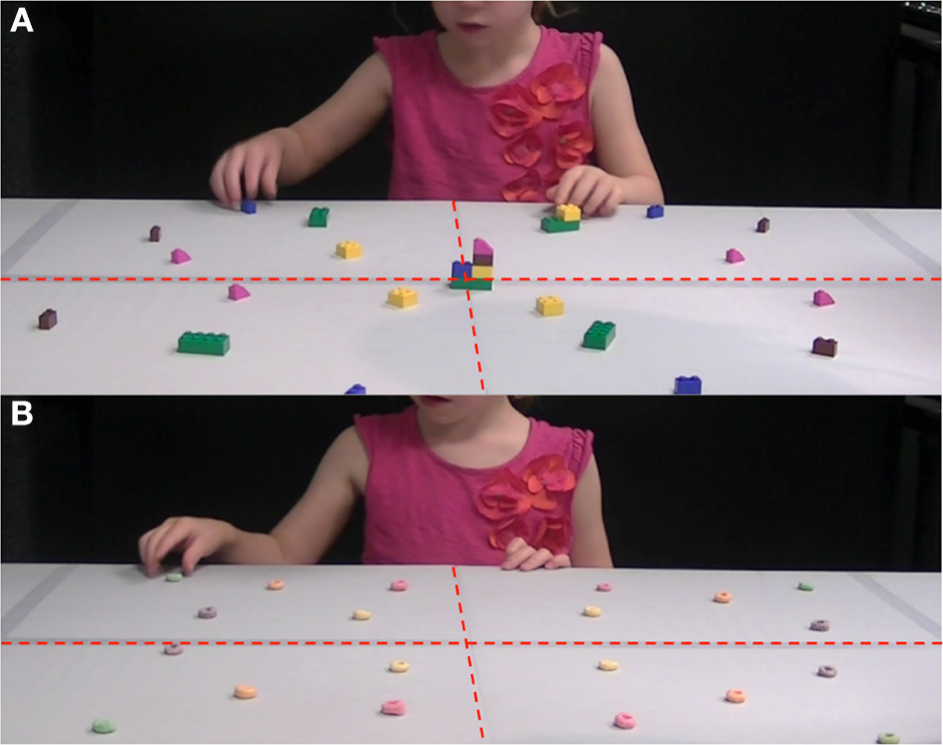
**The picture illustrates the workspace used by children in the grasp-to-construct (A) and the grasp-to-eat (B) tasks**. The tabletop was notionally divided into four quadrants of equal dimensions (lines were not visible). Four identical sets of five blocks or Froot Loops^®^ were placed on the tabletop—one set in each quadrant in near-mirror image placements. Within a set, items were unique in shape or color.

Children were given two grasping tasks: a grasp-to-construct and a grasp-to-eat (see Supplementary Materials for the videos of the tasks). In both tasks, the child was asked to sit and face a table with a workspace covered in Lego® blocks or in Froot Loops® (see Figure 2). The child was not given any instruction as to which hand to use, in either task. In the grasp-to-construct task, the child was required to replicate four pre-made models. Each one was comprised of one set of pieces (the same set placed in each of the four unmarked quadrants of the workspace—right-near, right-far, left-near, left-far, see Figure 2); thus, models contained the same pieces but in unique configurations. Within each age group, all children received the same four models, in the same order. The four sets of pieces on the table were placed in near-mirror image positions relative to one another, so that there was an equal opportunity to choose pieces from either side of space when completing the models. Individuals in the younger group (4–5 years old) sat at a table with a workspace 60 cm deep × 80 cm wide. These children encountered a total of 20 pieces on the tabletop; each of the four quadrants and four models contained the same set of five pieces. The older group (8–9 years old) sat at a table with a workspace 70 cm deep × 122 cm wide. These children encountered a total of 40 pieces (each quadrant and model contained the same set of 10 pieces). Once seated, the experimenter explained to the child that the object of the “game” was to make a model that looked just like the experimenter's model (see Gonzalez et al., 2014 for a similar description of the task). The experimenter pointed out a pre-made model, placed across from the child at the far end of the block array, aligned with the child's midline (see Figure 2A). Children in the older age group only, were asked to complete the replica as quickly as possible. Children were allowed to pick up the original model at any point during the task, and manipulate it in any way to understand its configuration. However, models were designed to be fully understood from a straight-on viewing angle. Once the first replica was completed, the experimenter removed the replica and replaced the first model with the next (in the same position). At the onset of the second trial, three sets of pieces were still available on the tabletop. After completion of all four replicas, all pieces on the table-top were used.

The grasp-to-eat task was always administered immediately after the child completed the grasp-to-construct task. The child was asked to remain seated at the same workspace, and the experimenter placed 20 Froot Loops® on the table (each unmarked quadrant contained five loops—one of each color). Again, items were placed on the table in near-mirror image positions relative to one another to ensure that they were equally accessible from the right or the left side (see Figure 2B). The experimenter then explained that she would call out colors (purple, green, pink, orange, or yellow) one at a time, and that the child should, upon hearing a color, reach out and grasp one matching loop as quickly as possible, then eat it. Once the food item was eaten, the experimenter would call out the next color. In this manner, the experimenter read out a list that contained four repetitions of each color (pseudorandomized and consistent across participants), totaling 20 requests.

In each task, participants' frequency of right hand use was calculated by scoring number of right hand grasps then dividing that number by total grasps (either 20 or 40 in the grasp-to-construct task; 20 in the grasp-to-eat task).

## Results

### Speech

#### Analysis of variance

A One-Way ANOVA showed a significant main effect of age on “s”–“sh” differentiation [*F*_(1, 34)_ = 8.4; *p* < 0.01]. As expected, older children displayed better differentiation between the two sounds suggesting that they produce the two sounds with greater articulatory distinctions with more tongue/lip displacement. Such a result is consistent with previous research reporting that the mastery of the two sounds continues in school-aged children (Sander, 1972; Smit et al., 1990).

#### Correlation

Chronological age to the day and “s”–“sh” differentiation were entered in the correlation. As the analysis of variance suggested, there was a significant positive correlation of chronological age with “s”–“sh” differentiation [*r*_(35)_ = 0.502; *p* < 0.01]. The older the child, the greater the distance between the two sounds.

### Grasping

#### Analysis of variance

A One-Way ANOVA on the frequency of right hand use during the grasp-to-construct task revealed no main effect of age [*F*_(1, 34)_ = 1.94; *p* = 0.17]. Similarly, there was no main effect of age on the grasp-to-eat task [*F*_(1, 34)_ = 0.8; *p* = 0.37]. Therefore, the data was collapsed across age. Right hand preference was greater [*t*_(34)_ = −2.54; *p* < 0.02] for the grasp-to-eat (73.85 ± 4.4) vs. the grasp-to-construct (63.8 ± 2.2) task.

#### Correlation

Chronological age to the day and right hand use for grasping in the grasp-to-construct and grasp-to-eat were entered in the correlation. There was no significant correlation between chronological age and either of the grasping tasks (*p* > 0.1). There was, not surprisingly, a significant correlation for right hand use in the two gasping tasks [*r*_(35)_ = 0.471; *p* < 0.01]. The more the right hand was used to pick up the blocks the more it was used for grasping the food items.

### Speech and grasping

#### Correlation

**Figure 3:**
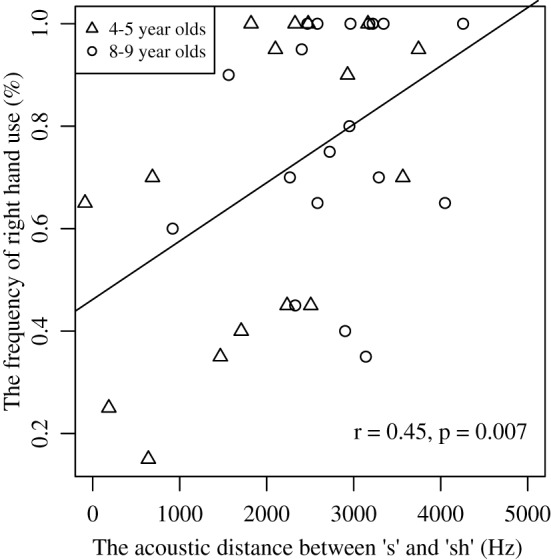
**The graph depicts the relationship between percent right hand use in the grasp-to-eat task and the “s”–“sh” acoustic distance for all children (4–5 and 8–9); in other words the relationship hand for grasping and speech**. A significant positive correlation was observed (*r* = 0.45, *p* = 0.007), indicating that the more the right hand was used for grasping the food items, the better s–sh acoustic differentiation.

Interestingly, there was a positive correlation between “s” and “sh” differentiation and right hand use in the grasp-to-eat task [*r*_(35)_ = 0.45; *p* < 0.01]. The greater the differentiation between the sounds, the more the child used the right hand to pick up the Froot Loops® (see Figure 3). The correlation between “s” and “sh” differentiation and right hand use in the grasp-to-construct task, however, was not significant (*p* = 0.55).

To further explore the relationship between sound differentiation and lateralization of grasp-to-eat, we ran separate correlations for the two age groups. For the young group we found the correlation to be significant [*r*_(17)_ = 0.527; *p* < 0.05]. Again, the greater the distance between the two sounds, the more the child used the right hand for grasping the food. For the older group the correlation was not significant [*r*_(18)_ = 0.230; *p* = 0.35].

### Age, speech, and grasping

#### Regression analysis

Does age or handedness best predict “s”–“sh” differentiation?

Chronological age and hand use for the grasp-to-eat and the grasp-to-construct tasks were entered as factors, with “s”–“sh” differentiation as the dependent measure. The model accounted for 37.0% of the variance, and it was significant [*F*_(3, 31)_ = 7.653; *p* < 0.001]. An examination of the coefficients showed that chronological age and right hand use for grasp-to-eat were both significant predictors of “s”–“sh” differentiation (see Table 1).

**Table 1:** **Results of the regression analyses**.

**Dependent measure**		**Coefficients**							
		**Unstandardized coefficients**		**Standardized coefficients**				**Correlations**	
		***B***	**Std. error**	**Beta**	***t***	**Sig**.	**Zero-order**	**Partial**	**Part**
“s”–“sh” differentiation	Chrono-age	0.66	0.19	0.48	3.41	**0.002**	0.50	0.52	0.46
	RH-Use blocks	−19.23	12.52	−0.24	−1.53	0.13	0.10	−0.26	−0.20
	RH-Use food	18.52	6.07	0.47	3.05	**0.005**	0.44	0.48	0.41
RH-Use food	Chrono-age	−0.002	0.006	−0.06	−0.33	0.73	0.17	−0.05	−0.05
	“s”–“sh” differentiation	0.012	0.005	0.47	2.58	**0.014**	0.44	0.41	0.41

*Note the relationship between right hand use in the grasp-to-eat (RH-Use food) and “s”–“sh” differentiation. The bold values are the significance*.

Furthermore, there was a reciprocal relationship between right hand use for grasp-to-eat and “s”–“sh” differentiation. When entering vocal differentiation and chronological age as factors in a model to predict right hand use for grasping for food, the model accounted for 14.9% of the variance and it was significant [*F*_(2, 32)_ = 3.97; *p* < 0.05]. The coefficients revealed that “s”–“sh” differentiation was a significant predictor of right hand use in the grasp-to-eat whereas chronological age was not (see Table 1).

## Discussion

The purpose of the present study was to investigate the possible relationship between motor performance and speech articulation in normally-developing children. To this end, children of two different ages (4–5 and 8–9) completed two grasping tasks and a test of speech articulation. Analyses performed on the grasping tasks were on hand use. Children were required to pick up a Froot Loop® to bring it to the mouth for consumption (grasp-to-eat), or a Lego® block to build a model (grasp-to-construct). For the speech articulation test, the measure was the acoustic distance between the “s” and “sh” sounds as the child produced the names of objects (containing these sounds) when they appeared on a computer monitor. The results showed that in the younger group, the more the child used the right hand for grasping food, the greater the distance between the two sounds. For the older group the correlation was not significant. This finding is not surprising, as studies have shown that the acquisition of the “s” and “sh” sounds begins at 3 years of age and is mastered by 7 years of age (Sander, 1972). The older group in this study was well-beyond this age. However, the regression analysis with all children included showed that hand use for grasping food items predicted the degree of speech differentiation. The more the child used the right hand for picking up Froot Loops®, the greater the distance between the “s” and “sh” sounds. This relationship was a reciprocal one. In other words, there appears to be an intimate relationship between lateralization for hand use during grasping-to-eat and the refinement of speech articulation. We propose a scenario wherein speech evolved from the grasp-to-mouth action for feeding behavior.

Evidence from developmental literature has provided support for the intricate relationship between the motor system and speech and language. Babies who experience difficulties latching during nursing are known to have a higher incidence of language delays (McFarland and Tremblay, 2006; Adams-Chapman et al., 2013). Children with specific language impairment (SLI) also present with motor deficits (e.g., Bishop, 2002; Zelaznik and Goffman, 2010; Finlay and McPhillips, 2013; Didonato Brumbach and Goffman, 2014). For example Finlay and McPhillips showed that 9–10 year old children with SLI were significantly worse than normally-developing children on measures of fine motor skill (posting coins, threading beads, and tracing). Furthermore, there is also evidence of lifelong visuomotor impairments in individuals with developmental stuttering (Jones et al., 2002). Taken together this evidence strongly suggests that language and motor system development is not independent.

Consistent with previous research (Sacrey et al., 2013) we found that right hand preference was greater for the grasp-to-eat than for the grasp-to-construct task in children. This finding supports the argument that there are fundamental differences in the lateralization of brain networks supporting actions that, while seemingly similar, differ in ultimate intent (Armbruster and Spijkers, 2006; Flindall and Gonzalez, 2013). More importantly, only the grasp-to-eat task was associated with speech differentiation abilities. This is an intriguing finding and suggests that lingual gesture and the grasp-to-eat movement (but not grasp-to-construct) share a common mechanism of action. Research has demonstrated a link between hand-to-mouth actions and vocalizations (Fogel and Hannan, 1985) and recently the hand-to-mouth action has been touted as an evolutionary stepping-stone to language (Corballis, 2003, 2009; Gentilucci and Corballis, 2006; Gentilucci and Dalla Volta, 2008). Given the likelihood that the refinement of the hand-to-mouth movement primarily subserved feeding (Macneilage et al., 1987) one would expect (as found) a relationship between language and the grasp-to-eat, but not grasp-to-construct, task.

In his frame/content theory of evolution of speech production, MacNeilage (1998) proposed that speech might have evolved from the repetitive movements involved in mastication. As stated by Walker (1998): “No one can disagree that the articulatory organs (tongue, jaws, lips, larynx) are also used in eating.” There is accumulating evidence showing a synergistic relationship between actions that involve the hand and actions that involve the mouth. For example it has been shown that grasping or observing grasps of two different sized fruits (apple and cherry) differentially influence voice spectra depending on the size of the fruit. When grasping or observing a grasp for an apple there was an increase in both the opening of the lips, and formant 2 (F2; which is related to tongue position) of the vowel “a” while producing the syllable “BA” (Gentilucci et al., 2004; see Gentilucci and Corballis, 2006 for a review). These results led the authors to hypothesize that evolutionarily, grasp observation might have been associated with the priming of mouth movements (for chewing, swallowing) that would later support speech (Gentilucci and Corballis, 2006). Our finding that hand preference for the grasp-to-eat action is a reliable predictor of the proficiency of speech articulation provides support for the speculation that the hand to mouth action, which developed for ingestion, may also subserve vocal communication (Gentilucci et al., 2009; Flindall and Gonzalez, 2013). Moreover, the grasp-to-eat action has been shown to be lateralized to the left hemisphere (Flindall and Gonzalez, 2013, 2014; Flindall et al., 2014). Specifically they have shown a right hand advantage in the kinematics of grasp-to-eat/hand-to-mouth actions that is absent from grasp-to-place actions. When grasping a small food item with intent to eat, participants produce tighter maximum grip apertures during the outgoing movement than when grasping the same item to place it in a receptacle near the mouth. This task difference in hand pre-shaping is predominantly lateralized to the right hand, regardless of a person's overall hand preference (Flindall and Gonzalez, 2013; Flindall et al., 2014). This evidence provides support for the notion that the lateralized hand-to-mouth system is a good candidate for the neural basis upon which hand preference for praxis (i.e., tool use and gesturing) and eventually language evolved (Flindall and Gonzalez, 2013; for review, see Corballis, 2003; Pulvermuller and Fadiga, 2010). Another possible contributor to the differences in kinematic parameters between seemingly similar grasping actions is the social intention of feeding between conspecifics (Ferri et al., 2011). In other words, it is possible that social interaction plays a role in shaping the relationship between grasp-to-eat actions and other socially-relevant behaviors (particularly language). In fact, a recent report has shown that in infants, hand preference for grasping can be temporarily modulated by social context. Six-month old babies who were verbally congratulated for using their left hand when grasping a toy, were more likely to continue using that hand for the duration of the experimental session (Morange-Majoux and Devouche, 2014). Taken together, these findings all support a theory of human motor system organized around a catalog of movements based on end-goal and intent, rather than mechanical requirements. Furthermore, they suggest that a social interactive context, wherein language and hand gestures (e.g., grasping-to-eat) are imbricated, provide a possible mechanism for our finding of a relationship between grasp-to-eat and speech articulation.

Given the relationship between hand-to-mouth actions and speech articulation it may be that an early means to detect potential disruption of language development is to screen for abnormalities in motor skill development. Furthermore, intensive language-action interventions have been touted as a means of improving language skills (for a review see Berthier and Pulvermuller, 2011). The results from our study support the intriguing possibility that training hand-to-mouth actions, specifically with the right hand, could enhance or accelerate maturation of at least some components of speech. This possibility of training hand-to-mouth actions is fundamentally different from the disputed practice of forcing a child to use their non-dominant right hand for school-work, particularly writing. An evaluation of a brief intervention (30 min a day maximum) in which children play with and grasp objects (not tools like a pencil or crayon) with the right hand is the intent. We are in the initial phases of evaluating such an intervention.

In conclusion, our study makes two unique contributions to the growing corpus of literature demonstrating a relationship between motor and language functions. We identified that the degree of lateralization for grasping predicts the maturity of the language production system in young, typically-developing children. Furthermore, we narrowed this relationship to hand-to-mouth actions specifically. We propose a scenario in which the neural substrates that afforded left hemisphere specialization for the grasp-to-eat action served as a foundation upon which speech articulation evolved.

### Conflict of interest statement

The authors declare that the research was conducted in the absence of any commercial or financial relationships that could be construed as a potential conflict of interest.
